# Aripiprazole for olanzapine-induced symptomatic hyper prolactinemia

**DOI:** 10.4103/0253-7613.62398

**Published:** 2010-02

**Authors:** Ashish Aggarwal, Manish Jain, Amit Garg, R.C. Jiloha

**Affiliations:** Department of Psychiatry, Indira Gandhi Medical College, Shimla, Himachal Pradesh, India. E-mail: drashish1980@yahoo.co.in; 1 Department of Psychiatry, Dr RML hospital, New Delhi, India; 2 Department of Psychiatry, Maulana Azad Medical College and G.B. Pant Hospital, New Delhi, India

Sir,

Aripiprazole is a novel atypical antipsychotic medication as it is a potent dopamine partial agonist and also acts as a partial agonist at serotonin (5-HT)_1A_ and as an antagonist at 5-HT_2A_ receptors.[1] It has been theorized that dopamine partial agonists may be able to stabilize the dopaminergic system without inducing a hypo dopaminergic state, thereby reducing the risk of side effects associated with pure blockade of dopamine receptors, especially extra pyramidal and endocrinological side effects. We hereby report a patient in whom addition of aripiprazole to ongoing treatment with olanzapine resulted in resolution of olanzapine induced hyper prolactinemia.

The patient: A 29-year-old married female suffering from schizophrenia since three years. Her illness was characterized by suspiciousness, muttering to self, violent, aggressive behavior and disturbed biological functions. There was no significant past or family history of any psychiatric or neurological illness. She was initially treated with risperidone and haloperidol, each in adequate dose and for adequate duration, without much response. Her family members did not agree to clozapine fearing that they would not be able to monitor the blood counts. Subsequently, she was started on olanzapine up to 20 mg per day and gradually started showing improvement in symptoms. She was maintaining well when she decreased the dose on her own and subsequently had exacerbation of her symptoms. As a result, olanzapine was increased to 25 mg per day. After about one month of this therapy, the patient started reporting breast tenderness with milky discharge from nipples. She also reported scanty menstruation. At this time, her serum prolactin level elevated and was at 47 ng / mL (normal: 1.5–19.0 ng / mL). Ancillary tests, including thyroid profile and brain computed tomography scan, were normal. In view of past non response to antipsychotic drugs, olanzapine was continued in the same dose and aripiprazole was added to olanzapine in the dose of 15 mg per day. After two weeks of this addition, her serum prolactin level was 27 ng / mL. After about a month of this, the patient's symptoms improved completely and her prolactin level was within the normal range (11 ng / ml). The patient has been maintaining well on this combination for the last six months.

There are similar reports, of combining aripiprazole with risperidone and with haloperidol to offset hyper prolactinemia.[2,3] Paradoxically, there are also reports of aripiprazole causing galactorrhea.[4,5] and not being effective in treatment of hyper prolactinemia associated with antipsychotic medication.[6]

Although a switch to aripiprazole is an understandable and probably useful strategy, it might not be clinically useful to switch to another antipsychotic when a patient has responded to the medication. Our case did not respond to a previous trial of risperidone and haloperidol and it was not clinically feasible to stop or taper olanzapine. Perhaps these apparent inconsistencies may be reconciled by considering that aripiprazole is a partial agonist, thereby it may act as either an agonist or an antagonist depending upon the conditions. The partial agonist property of this compound means that in the presence of dopamine hypo activity, as induced by olanzapine, aripiprazole functions as a dopamine agonist with roughly 30% intrinsic activity at postsynaptic receptors,[1] restoring tonic inhibition to anterior pituitary lactotrophs.

Spontaneous prolactin decline in this case would be unlikely because the time since olanzapine exposure was short. Our report suggests that aripiprazole might be useful as an add-on therapy in patients who develop hyper prolactinemia as a side effect to another antipsychotic without affecting the clinical outcome.
